# Supplementary material for: Protocol for a magnetic resonance imaging study of participants in the fever RCT: Does fever control prevent brain injury in malaria?
Source: PLoS One. 2024 Apr 19;19(4):e0294823. doi: 10.1371/journal.pone.0294823 (PMC11029645; doi:10.1371/journal.pone.0294823)
Supplement: S1 File — (PDF) [file pone.0294823.s002.pdf]

An MRI Ancillary Study of Malaria FEVER RCT
NIH Grant No. R01NS111057
Principal Investigator – Gretchen L. Birbeck

Institutions: University of Rochester (UR), Rochester, NY, USA
Queen Elizabeth Central Hospital (QECH), Blantyre, Malawi
University Teaching Hospital's Children's Hospital (UTH), Lusaka, Zambia
Chipata Central Hospital (CCH), Chipata, Zambia
Michigan State University (MSU), East Lansing, MI, USA

Investigators: Gretchen L. Birbeck, UR & UTH
Michael Potchen, UR & Lusaka Apex Medical University
Sam Kampondeni, UR and Mpingwe Clinic (Blantyre)
Karl Seydel, QECH & MSU
Yamikani Chimalizeni, Malawi College of Medicine and QECH
Manoj Mathews, UTH
Musuku Mwenechanya, UTH
Suzanna Mwanza, CCH
Colleen Hammond, MSU
John Langfitt, UR

1. EXECUTIVE SUMMARY

Despite eradication efforts, ~400,000 African children sustained brain injuries as a result of CNS malaria in 2016. A higher maximum temperature (Tmax) during the acute malaria infection is an established risk factor for neurologic sequelae and a randomized controlled trial (RCT) of aggressive antipyretic therapy with acetaminophen and ibuprofen being conducted in Malawi and Zambia began enrollment in 2019 (R01NS102176). In this clinical trial, the primary outcome is Tmax during the acute infection. However, the antipyretic therapies used in this RCT may offer neuroprotective effects without affecting Tmax--for example, neuroprotection through anti-inflammatory mechanisms. In **An MRI Ancillary Study of Malaria FEVER RCT**, we propose to use neuroimaging in the context of the RCT to further evaluate the potential neuroprotective effects of aggressive antipyretic therapy for CNS malaria and explore possible mechanisms for these effects. Comparing children allocated to aggressive antipyretic therapy vs. usual care on the prevalence of structural brain abnormalities after recovery from CNS malaria will facilitate the evaluation of non-fever pathways for neuroprotection. Brain MRIs will be obtained in children enrolled in the RCT at 1- and 12-months post recovery. Analyses will be completed comparing the odds of having any structural injury based upon RCT treatment allocation and based upon (Tmax) stratified by treatment allocation to assess changes specifically related to response to therapy in terms of fever reduction. Potential mechanisms of aggressive antipyretic-related injury will be evaluated including assessments for treatment-related CNS bleeds. Neuroimaging is a well-established, valid proxy for neurological outcomes after brain injury including in pediatric CNS malaria. Adding this MRI ancillary study to our fever RCT may elucidate mechanisms of treatment-associated injury and allow for early identification of neuroprotection from aggressive antipyretic use that would otherwise require long-term follow-up for cognitive and behavioral assessments. This study will provide critical insights that could inform future neuroprotective studies of malaria that might incorporate imaging to optimize study design.

2. MAIN OBJECTIVES

This prospective observational study will seek consent from parents of children previously enrolled in the Aggressive Antipyretics in CNS Malaria: A Randomized Controlled Trial Assessing Antipyretic Efficacy and Parasite Clearance study (NIH Grant No. R01NS102176; ClinicalTrials.gov NCT03399318) to obtain neuroimaging and neurodevelopmental and behavioral outcomes data on their child. The imaging and evaluations for this observational study will occur after the child has recovered from the acute malaria infection and has otherwise completed the RCT intervention and safety evaluations. For some, consent will be sought months *after* the RCT-related activities for the child are completed. As such, this is an independent observational study. However, since the findings may offer relevant outcomes data for the RCT, data obtained

in this observational study will be shared with the RCT's biostatistician for inclusion in all interim analyses and review by the RCT's Data Safety Monitoring Board (DSMB).

Specific Aims
Aim 1: To compare the prevalence of brain injury in theaggressive antipyretic (AA) vs. usual care (UC) groups of the FEVER RCT.

Aim 2: To compare the prevalence of specific structural injuries amongchildren who received AA therapy vs. UC with propensity score adjustment.

Aim 3 Safety assessments.

3. BACKGROUND AND INTRODUCTION

CNS malaria, meaning *P. falciparum* parasitemia with neurologic symptoms including impaired consciousness and/or seizures,[1-3] continues to be a major cause of neurodisabilities in African children. Brain injury with subsequent neurologic sequelae occur in 1/3rd of children who survive CNS malaria. Newer antimalarial medications rapidly clear peripheral parasitemia and improve survival, but mortality remains high at 12-25% with no associated decline in post-malaria neurologic injury.[4] Common sequelae include epilepsy, gross motor deficits, deafness, blindness, cognitive impairment, and behavioral disorders.[1-3, 5-8]

In our prior Blantyre Malaria Project Epilepsy Study (BMPES), a higher maximum temperature (Tmax) during the acute infection was associated with a greater risk of post-malaria neurologic sequelae. Children who developed epilepsy had a mean Tmax of 39.4°C vs. 38.5°C in those who did not develop epilepsy (absolute difference of 0.9°C; p=0.01). In addition, a higher Tmax predicted subsequent behavioral disorders (39.2°C vs. 38.7°C; absolute difference 0.5°C; p=0.04).[5] These data are biologically consistent with the substantial body of evidence that fevers contribute to secondary neurologic injury in the setting of stroke, meningitis, anoxia, hypoxia, and trauma.[9-13] In the U.S., induced hypothermia is now standard-of-care for post-anoxic[14] and neonatal hypoxic-ischemic encephalopathy[15-18] and fever-reduction is an important aspect of neurocritical care.[19-22] Malaria-induced fevers can be extremely high. Fifteen percent of the BMPES cohort experienced at least one temperature of ≥ 40.0°C (104.0°F). The age-related susceptibility to pediatric malaria also substantially overlaps with the age-related phenomena of febrile seizures. Malarial seizures are often complex, multifocal, and prolonged with *status epilepticus* being common.[5, 7, 23, 24].

In 2019, a randomized controlled trial (RCT) of aggressive antipyretic therapy for CNS malaria launched in Malawi and Zambia. This RCT compares prophylactic, scheduled acetaminophen and ibuprofen for 72 hours after presentation with CNS malaria to usual care with the primary outcome being Tmax as "proof-of-concept" for potential neuroprotective efficacy of aggressive antipyretic therapy in CNS malaria. **This observational MRI Ancillary study will obtain neuroimaging and neuropsychiatric outcomes in children enrolled in the RCT.**

Rationale
While a higher Tmax is associated with adverse neurologic outcomes in CNS malaria, causality is not directly established. CNS malaria is a highly inflammatory process[25, 26] and inflammation-mediated injury is a likely pathway for malaria-associated brain in this population particularly for epilepsy development. The aggressive antipyretic regimen should offer anti-inflammatory benefits as well. Neuroimaging after recovery will facilitate identification of benefits associated with the aggressive antipyretic therapy that are mediated by non-fever pathway including reduced inflammatory factors. Our research team recently published findings from a 1-year follow up study of children who survived cerebral malaria compared to age-matched controls, which confirmed high rates of cognitive and behavioral impairments (53% at one year) in the malaria group. Importantly, neurodevelopmental disabilities at 12 months were associated with structural brain lesions on MRI at 1-month including severe atrophy and multifocal abnormalities.[27] As such, utilizing neuroimaging findings captured at 1- and 12-months post recovery as an outcome in the RCT will also allow us to assess for potential

neuroprotective benefits from aggressive antipyretics in CNS malaria that are not necessarily mediated by fever.

Neuroimaging at 1- and 12-month post recovery will also facilitate additional safety assessments. Though frank CNS bleeds are not associated with pediatric cerebral malaria,[28] end vessel microhemorrhages are seen post mortem[29, 30] and have been identified on brain MRIs in case reports of cerebral malaria.[31, 32] Using imaging sequences that identify ferromagnetic substances, we will evaluate for evidence of any hemorrhages including parenchymal microhemorrhages and compare the presence and characteristics of any CNS bleeding between the two arms of the RCT. Given the antiplatelet effects of ibuprofen, increased risk of bleeding is conceivable in the aggressive antipyretics arm. Data from this MRI Ancillary study will be provided to the RCT's DSMB for the scheduled interim analysis.

4. ADMINISTRATIVE ORGANIZATION

All patient-contact will occur in Zambia and Malawi where the RCT is ongoing. All data transmitted or conveyed beyond the country of enrollment will be de-identified.

<u>Study Sites:</u>
University Teaching Hospitals-Children's Hospital, Lusaka, Zambia
Chipata Central Hospital, Chipata, Zambia
Queen Elizabeth Central Hospital, Blantyre, Malawi

Study sites without patient contact or access to data with private health information include—
University of Rochester, Rochester, NY, USA
Michigan State University, East Lansing, MI, USA

<u>Oversight plan for non-UR affiliated US investigator:</u>

While in Malawi, the MSU PI is responsible for overseeing subject recruitment, data collection and imaging at Queen Elizabeth Central Hospital. As part of data cleaning, they will also have access to data with patient identifiers. Our office takes responsibility for the required IRB reports (the University of Rochester's Research Subjects Review Board, The University of Malawi's College of Medicine Research Ethics Committee, and the University of Zambia's Biomedical Research Ethics Committee) within Malawi and Zambia. Regular communications between the MSU PI, Local Safety Monitors (LSM) and the PI ensure that data collected and contact information is up-to-date.

No human subjects contact will occur at any of the US-based sites. All data exported to the US is de-identified prior to transfer. All RSRB determinations regarding this work will be shared with the non-affiliated researchers in a timely fashion.

Data storage and analysis will also take place using UR servers but only de-identified data will be externalized. All data transmitted to UR will be de-identified.

5. STUDY DESIGN

<u>To compare the prevalence of brain injury in the aggressive antipyretic vs. usual care (UC) groups of the Fever RCT</u>
Children will undergo brain MRIs and neurodevelopmental and behavioral evaluations at 1- and 12-months after their acute malaria illness or as soon as possible among those children enrolled in the Fever RCT prior to the initiation of this MRI ancillary study. Two radiologists, blinded to treatment allocation and neurologic status, will independently review images and capture data using NeuroInterp.[33] For children who are unable to undergo imaging without deep sedation, neurodevelopmental & behavioral evaluations will be used to identify those with brain injury.
We hypothesize that children who receive AA therapy during CNS malaria will have lower odds of brain injury than those receiving UC.

To compare the prevalence of specific structural injuries among children who received aggressive antipyretic therapy vs. usual care therapy in the Fever RCT. Specifically, we will compare

a. Overall atrophy by brain volume scoring[34] (atrophy being a brain volume score of 1-2 vs ≥3)
b. Gliosis by Fazekas score[35] (0, 1, 2 or 3)
c. The presence of regional gliosis or atrophy in the following regions
    1. Cortical (present/absent)
    2. Deep grey (present/absent)
    3. Corpus callosum (present/absent)
    4. Posterior fossa (present/absent)

A priori pathophysiologic attributions are as follows:
-If focal cortical abnormalities are lower for the AA treatment group, we will conclude that the mechanism(s) included seizure control/prevention
-If abnormalities are less common in the deep grey regions for the AA treatment group, we will attribute this to benefits of reduced sequestration in the deep vascular beds
-If there is less diffuse atrophy or overall gliosis for the AA treatment group, we will attribute this to reductions in increased intracranial pressure

Safety assessments
a. In Zambia where standard imaging with GRE sequences is available, we will compare the prevalence of blood products in the AA vs UC groups. If present, blood products will be further characterized by age of blood relative to participation in the Fever RCT and blood volume based upon number and size of foci.
b. The prevalence of CKD based upon blood creatinine and urine ACRs will be compared in the AA vs UC groups

5.1. SUBJECT POPULATION
All children enrolled in the Malaria RCT who survive to discharge are eligible for enrollment. Inclusion and exclusion criteria for the malaria fever study are outlined below.

6. INCLUSION AND EXCLUSION CRITERIA
All children enrolled in the malaria RCT who survive to discharge are eligible for inclusion. The malaria FEVER RCT inclusion and exclusion criteria are detailed below.

Inclusion Criteria for RCT
Age 2-11 years (24 to 132 months)
Evidence of P. falciparum malaria infection by peripheral blood smear or rapid diagnostic test
CNS symptoms associated with malaria.

CEREBRAL MALARIA: Impaired consciousness with a Blantyre Coma Score (BCS) ≤2 in children under 5 years or a Glasgow Coma score (GCS) ≤10 in children ≥5 years OR CNS MALARIA: Complicated seizure(s), meaning prolonged (>15 minutes), focal or multiple; or impaired consciousness or other evidence of impaired consciousness (confusion, delirium) without frank coma (BCS>2, GCS =11-14)

Exclusion Criteria for RCT
Circulatory failure (cold extremities, capillary refill > 3 seconds, sunken eyes, ↓ skin turgor)
Vomiting in the past 2 hours
Serum Cr > 1.2 mg/dL
A history of liver disease
Jaundice or a total bilirubin of >3.0mg/dL
A history of gastric ulcers or gastrointestinal bleeding

A history of thrombocytopenia or other primary hematologic disorder
Petechiae or other clinical indications of bleeding abnormalities
A known allergy to ibuprofen, acetaminophen, aspirin or any non-steroidal medication
Any contraindication for nasogastric tube (NGT) placement and/or delivery of enteral medications

7. RECRUITMENT METHODS

Before recruitment into this observational study will have begun, consent for future contact regarding possible research participation in subsequent relevant studies was sought from the parents/guardians of children enrolled in the Malaria FEVER RCT at the time of discharge. This process was approved by the appropriate ethical and institutional review boards. A list of those who consented by study ID will be cross-referenced to the contact details including address and cell phone numbers maintained in the study pharmacies. These parents/guardians will be contacted to seek consent for enrollment in this observational study.

In addition, children being enrolled in the ongoing RCT will be identified as potential participants when they have been stabilized and regained consciousness. Study Nurses working on the ward where the children are admitted will then approach the parent/guardian for consent prior to discharge.

8. CONSENT PROCESS

For children newly admitted for the RCT study, nurses on the ward where the children are admitted will approach the parent/guardian for consent. For children recently admitted but already discharged whose parents consented to be contacted regarding future studies, phone calls will be placed and a brief overview of the study will be detailed. If the parent/guardian is willing to consider enrollment, they will be invited to come to the hospital for a full discussion and the consent process detailed below.

Consent will take place in a private setting. Staff obtaining consent are fluent in the local languages and expert in conducting the informed consent discussion. All have Human Subjects Protection and Good Clinical Practice certifications. The consent process will be conducted in the preferred language of the parent/guardian. Only local staff will be involved in this process due to the geopolitical/post-colonial nature of this setting. The parent/guardian will be given whatever time is needed to consider whether they are willing to have their child enrolled including opportunities to discuss with other family members. All parents/guardians who consent will be provided with signed copies of the consent forms. For those parents/guardians who are unable to sign due to literacy limitations, a thumb print will be obtained and someone who is not associated with the study but is literate in the parent's/guardian's preferred language will be present during the consent process and will sign as a witness. At the time of the return visit for evaluations, for children 7 years or older, if in the parent's/guardian's assessment is that the child has the capacity to comprehend the nature of the request, assent will also be sought.

Consent and assent forms will be developed in English and the applicable local languages. Forward- and back-translation will be undertaken by expert local staff with extensive experience in this regard.

9. STUDY PROCEDURES

At 1- and 12-months post admission for malaria or as soon as possible if the index illness was > 12 months prior to the launch of this observational study, enrolled children will undergo neuroimaging, the Neurological Examination for Subtle Signs (NESS) if 7 years or older, the WHO Epilepsy Screen and a single question behavioral assessments. In addition, depending upon age at the time of the evaluation they will undergo the Malawi Developmental Assessment Tool (MDAT) or the Kaufman Assessment Battery for Children (KABC). The MRI scan will require ~30 minutes and the remaining assessments ~1-1.5 hours. If a child is more than 1-month post discharge are the time of consent, the first assessments will be obtained as soon as is feasible. If any clinically relevant findings are identified, these will be discussed with the parent/guardian and the appropriate referral for care will be made.

MRI images will be reviewed by the technician at the time of the scanning and any findings of concern that might warrant gadolinium administration will be reviewed by one of the study radiologists in real time. This can

be done through the Baynes.net system in place for review and/or through sharing of the key images of concern to the technicians. This should allow for gadolinium administration when the child is initially imaged. However, if on review of an MRI that the technical staff had no concerns about the radiologists feels gadolinium is warranted for clinical care, the patient’s family will be notified and all the same financial and logistical support will be provided for this repeat image. This data will be used for research purposes if such images are recommended for clinical care.

10. DISSEMINATION
Findings from this work will initially be presented at appropriate local venues before broader dissemination. For example, UTH’s Neurology Research-in-Progress weekly meeting, Zambia’s Annual National Health Research Conference, the Wellcome Trust’s Cutting Edge weekly meeting, and/or Malawi’s College of Medicine Research Dissemination Day. With approvals from Zambia’s National Health Research Authority and the Malawi Ministry of Health, findings will then be submitted to peer reviewed journal(s) for publication and wider dissemination. Copies of published papers will be submitted to the associated IRBs, the College of Medicine Library in Malawi, and the Zambian National Health Research Authority.

11. RISKS TO SUBJECTS
Risks include loss of privacy, annoyance at the study assessments and adverse effects from sedation. MRI-specific risks are largely associated with implanted devices which are not in use in the study populations.

The study assessments including the neuroimaging are expected to take less than 2 hours. Regarding sedation risks--in our experience, children as young as 6 years are able to tolerate clinical MRIs without sedation with a parent in the scanning room to comfort and encourage them. Chloral hydrate has been routinely used for sedation for EEG and MRI at QECH in Malawi since both technologies became available over a decade ago. No serious adverse events have occurred in relation to chloral hydrate use in Malawi for this purpose. An estimated 6% of Malawian children given chloral hydrate develop a paradoxical hypervigilance sometimes with irritability and agitation lasting 4-6 hours. Nausea, vomiting and an allergic reaction are listed side effects of chloral hydrate though these have not been seen during their clinical use in children in Malawi over the last decade. Children with a history of an allergy to or adverse reactions to chloral hydrate will not undergo sedation with this agent. Children with a history of a paradoxical response to other sedatives will not receive chloral hydrate. Side effects for chloral hydrate are dose related. Although standard protocol for clinical use of chloral hydrate indicates repeated dosing if needed, no repeat dosing will be undertaken in this study. Due to the increasing concerns about tissue deposition of gadolinium, gadolinium will only be used if non-contrast imaging identifies a finding that clinically requires gadolinium for further assessment. By this we mean that is will not be administered for the purposes of this study but should an abnormality be identified that the radiologist feels requires gadolinium for clinical evaluation, study resources will be provided to ensure the additional imaging and gadolinium are provided.

We have been able to harmonize sedation protocols across sites to begin with melatonin and, if needed, add chloral hydrate. Administration of either medication is not complex (see Addendum II of Protocol V5.0). Melatonin, a dietary supplement, is safe and effective for sedation in sub-Saharan Africa.[36] Any interaction between melatonin and sedatives such as chloral hydrate are classified as minor and do not require adjustments in medications dosing. All children will be NPO for 4 hours prior to MRI in case any sedation is needed with snacks provided at the completion of the study/

In Zambia, melatonin is the standard sedative used for pediatric procedures such as EEG, but they have less experience with chloral hydrate. In addition, the imaging facilities in Zambia are outside of the pediatric hospital with limited support for managing adverse events related to sedation. As such, when chloral hydrate is used in Zambia, an anesthetist will administer the agent and be available until the child is fully awake.

In Malawi, they are less familiar with melatonin and study staff will receive in-service training regarding potential side effects which include dizziness, headache and nausea. These are self-limited and likely uncommon--Zambian providers indicated these are not events they have had reported to them when using

melatonin in children for EEG. In Malawi, chloral hydrate has long been the standard management for sedation for EEG and MRI and imaging occurs within the pediatric hospital with ample experienced staff in attendance so no additional monitoring measures beyond the clinical standard will be instituted.

Should the child experience any adverse effects from sedation, a study clinician will evaluate them and determine if further management if needed. Most anticipated side effects are self-limited and will resolve without intervention. If required, the clinician will provide analgesics for headache, anti-emetics for nausea, and oral rehydration solution for vomiting.

10. POTENTIAL BENEFITS TO SUBJECTS

Children identified as having neurologic sequelae based upon the evaluations (imaging and study instruments) will be referred for appropriate services that are available free of charge at both study sites. Children with structural lesions found on neuroimaging will be offered quarterly developmental screening assessments performed by our team for the duration of this study with the (MDAT/KABC, WHO Epilepsy Screening Questionnaire and the behavioral screen), to facilitate expedited care, if needed. Research dissemination meetings held with families of children who participated in our prior research conducted by this team identified this approach as favored by parents/guardians to help them in monitoring children at increased risk of neurologic sequela after recovery from cerebral malaria.

When children are evaluated for research purposes and problems are identified, it is an established practice for both the Zambia and Malawi-based teams to provide quarterly follow-up to facilitate expedited care if children in the study are not seen quarterly, but these additional evaluations are not for research purposes. Full reports of the MDAT and KABC are provided to the clinicians and therapists caring for the patient. The findings are also discussed personally with the parents by the study nurses and for those children who have problems identified a clinician will also review the findings with the parents and explain the reason that additional care and monitoring is being recommended. If the child is school age, a letter is provided to the school alerting the teacher that the child may have neurological problems involving behavior and/or cognition related to their severe malaria infection, that they are under medical care for this problem, and encouraging the teacher to allow the child to remain in school despite their challenges.

There is no precedence in this setting for special education in the public sector. Impaired children are usually just ejected from school and re-entry is challenging. Our goal in the communication to schools is to encourage the teachers to allow the children to remain in school. Providing full written reports of the MDAT and/or KABC would have little meaning to families or teachers in this environment.

11. COSTS FOR PARTICIPATION

Participants' and their families will not be required to cover any of the costs associated with participation. All study activities and procedures will be provided free-of-charge, as well as round trip transportation for the children and their parents/guardians for these follow-up assessments.

12. PAYMENT FOR PARTICIPATION

- In Malawi, for each study visit participants and their families will be reimbursed for the roundtrip travel costs for the child and guardian. They will also receive remuneration consisted with published guidelines which is MWK4500.[37]
- In Lusaka, for each study visit participants and their families will be reimbursed for the roundtrip travel costs for the child and guardian. They will also receive a total of ZMW260 for their time from work, food during the testing day and their child's completion of the assessments.
- In Chipata, for each study visit we will provide round trip transportation and overnight accommodation for the child and a parent/guardian to travel to Lusaka. They will also receive ZMW1060 for their time from work, food while traveling and the child's completion of the assessments.

13. SUBJECT WITHDRAWALS

Parents/guardians may withdraw their children from the study at any time.

14. PRIVACY AND CONFIDENTIALITY OF SUBJECTS AND RESEARCH DATA
To limit risk of loss of privacy, only the study ID number will identify all laboratory specimens, evaluation forms, reports, and other records that leave the sites. All paper records will be kept in secure study offices in locked filing cabinets. All computer entry and networking programs will be done using study IDs only. Study data using only study IDS will be store as follows: paper-based in the secure study offices at each site available only to study staff; neuropsychiatric outcomes Neuropsych and NeuroInterp data on the University of Rochester's Research Electronic Data Capture (REDCap)RedCap server, neuroimaging files in DropBox with secured folders accessible only to study staff. Study staff will assure that imaging data and scanned paper-based forms with graphical or pictorial data containing only the study ID and are saved in secured DropBox secured (i.e. non-public) folders. The study offices are secured rooms with lockable file cabinets. All computers are password- protected. DropBox folders containing de-identified data are non-public.

Clinical information will not be released without written permission of the participant's parent or guardian, except as necessary for monitoring by the appropriate institutional review boards (IRBs), the NINDS, the OHRP, the sponsor, or the sponsor's designee. If a family needs to be re-contacted for any reason (e.g. an imaging finding that is determined to warrant clinical follow-up), the study ID in this observational study will be cross referenced against the contact details for each child's family that is maintained as required in the study pharmacy for the RCT.

15. POSSIBLE CONSTRAINTS
The RCT has commenced and those patients enrolled to date will not be able to provide 1-month imaging. Public health concerns related to COVID-19 is anticipated to further delay this study. Fortunately, the chronic structural abnormalities that are seen with cerebral malaria-associated brain injuries are not typically reversible and should be seen even if children are imaged later than planned. Time from malaria infection to imaging can be controlled for in analyses.

16. DATA / SAMPLE STORAGE FOR FUTURE USE
De-identified imaging and neuropsychiatric outcomes will be stored for future use in secured DropBox folders and on the UR RedCap server. No data with identifiers is obtained or stored. Study IDs are used exclusively, but can be linked back to the subject if needed using the contact listing maintained in the study pharmacies for the RCT as required.

17. DATA AND SAFETY MONITORING PLAN
This is an observational study. Potential adverse events include breach in confidentiality and any injuries or inconveniences related to participation in the clinical assessments and/or imaging acquisition. Any adverse events identified will be reported to the appropriate institutional review boards.. Although not anticipated, should any Serious Adverse Events (SAE) occur during the conduct of this observational study, the Local Study Monitors serving the RCT will be asked to review with their assessments provided to the overseeing IRBs.

The children in this study have participated in an ongoing blinded RCT and their allocation either to aggressive antipyretic therapy or routine clinical care during their acute malaria infection remains concealed. As such, the findings from this observational study will be provided to the RCT biostatistician for analysis and inclusion in safety evaluations routinely conducted by the DSMB.

18. DATA ANALYSIS PLAN

The analysis of the presence of a brain injury (yes/no) will involve fitting a logistic regression model with RCT treatment allocation group as the factor of interest, country as a stratification factor, and a stratified propensity score[38] to account for the fact that the treatment groups will no longer be the original randomized groups due to participants refusing consent and loss-to-follow-up. The propensity score will be estimated using a logistic regression model with treatment group as the outcome variable and baseline covariate information that is thought to be relevant to predicting adverse neurological outcome. These covariates include disease severity (presence/absence of cerebral malaria), seizures prior to admission, admission temperature, admission creatinine, sex, and age. The estimated treatment group odds ratio, along with its associated 95% confidence interval and p-value, will be derived from this model. Similar analyses will be conducted for the other outcomes of interest including the presence of the specific imaging outcomes outlined in Aim 2, the presence of blood products; and the prevalence of chronic kidney disease based upon an ACR of >1.2. For the ordinal gliosis outcomes in Aim 2, a proportional odds logistic regression model will be employed; if the proportional odds assumption appears to be seriously violated, a partial proportional odds logistic regression model or a multinomial logistic regression model will be used instead.

The sample size considerations are based on the logistic regression analyses to be performed in Aim 1. Preliminary data indicate that 34% of pediatric CM survivors in Malawi have abnormal brain MRIs at 1 month or a KABC falling at least 2 standard deviations below the population norms for those over 7 years.[39] A sample size of 184 subjects (92 per group) will provide > 85% power to detect a reduction in the percentage of subjects with structural injury from 35% in the UC group to 15% in the AA therapy group, using a two-tailed test and a 5% significance level.

Addenda

Addendum I. Protocol Changes Made to Date

Protocol Version Changes	
Version 1.0 to 2.0	1. Removed plans for use of CT as contingency
Version 2.0 to 2.1	1. Reformatted for COMREC adding executive summary, dissemination plan and constraints
Version 2.1 to 3.0	1. Changed period for NPO before MRI sedation from 8 to 4 hours<br>2. Added specific amount of payment for Malawi participants
Note:<br>COMREC mandated that for their review and approval the protocol be adapted to their preferred format. This was done using Protocol version 3.0<br><br>UNZA BREC mandated that for their review and approval the protocol be adapted to their preferred format. This was done using Protocol version 3.0	
Version 3.0 to 4.0	1. Added oversight plan for non-UR US investigator
Version 4.0 to 5.0	1. Added Addendum 1 delineating all protocol changes from version 1.0 onward to be updated as needed<br>2. Revised running title in header to better reflect this is the MRI Ancillary and not the parent FEVER study protocol<br>3. Sedation protocol added as addendum 2<br>4. Instruments associated with the protocol were adjusted to add the single question behavioral screen and are now deemed v2.0<br>5. Consents and assents associated with protocol are now version 3.0
Version 5.0 to 5.1	1. Added further details on sedation protocols within both sites<br>2. Added details re management of any sedation adverse events<br>3. Added details re acute clinical review of MRI and use of gadolinium<br>4. Added details about services provided when abnormalities are identified in developmental assessments including communications with teachers<br>5. Added specifically that MRI will take ~30 minutes<br>6. The same detail re 30 minutes for MRI was added to the consent form. This protocol is associated with Consent version 3.1 and Assent version 3.0
Version 5.1 to 6.0	1. Add BRIEF to neurodevelopmental assessment instruments<br>2. Obtain creatinine and urine sample for dip and albumin: creatinine ratio to identify early signs of chronic kidney disease (CKD)<br>3. Swab to identify co-infection at time of malaria infestion with SARS-Cov-2

Version 6.0 to 6.1	Minor verbiage adjustments in protocol requested per RSRB for clarity only.
Version 6.1 to 7.0	1. Sedation clarifications<br>a. In Malawi, drop melatonin and allow snack<br>b. In Zambia, if imaging undertaken at Medland Hospital and sedation is needed, management for sedation is deferred to consultant anesthesiologist<br>2. At Chipata Central Hospital only, option for participation in expanded study of autism and epileptogenesis. This has a separate consent.
Version 7.0 to 7.1	<u>Amendments proposed</u><br>1. Changing of analysis plan, utilizing the propensity score adjustment and<br>2. aligning that change to slightly change the aims of the study<br><br><u>Rationale ;</u><br>bring clarity<br><br><u>Risks</u><br><u>No risks since no changes to the human subjects or enrolment process.</u>

<u>Addendum II. Dosing schedule for sedation</u>
Given the potential need for sedation, children will be NPO 4 hours prior to the scheduled MRI. Snacks will be provided at the completion of the MRI.

a. Melatonin

Provided in 3 mg chewable tabs
Time: 30 min – 60 min before MRI
Dose:
< 6 y/old: ½ tab (1.5mg)
>6 y/old: 1 tab (3 mg)
>10 y/old: 1 +1/2 tab (4.5mg)

If at 60-minutes post dosing the child is still too active for scanning, proceed to chloral hydrate. In Zambia, this requires administration by an anesthetist and rescheduling the procedure may be necessary. In Zambia, the anesthetist will remain with the child from the administration of chloral hydrate until the child fully regains consciousness (BCS 5/5).

b. Chloral hydrate

Provided in liquid form. Give 50mg/kg po 30 minutes before MRI. Do not repeat dose.

Addendum III.

Protocol Amendment version 5.1 to 6.0

<u>Amendment changes proposed:</u>
1. Add BRIEF (Behavioral Rating Inventory of Executive Function) for school and pre-school age children to assessment instruments f

2. Evaluate renal function at 1- and 12 months follow-up looking for any evidence of early chronic renal disease
    a. Creatinine by fingerprick point-of-service test
    b. Urine sample for dipstick evaluation and albumin/Cr ratio
3. Offer SARS-CoV-2 testing at enrollment which coincides with discharge from acute RCT admission to determine if the child had concurrent SARS-CoV-2 infection

Rationale

For adding BRIEF

Behavioral abnormalities are well-documented sequelae in children surviving cerebral malaria (CM).[5] Previous work with instruments developed for pediatric populations in the west, such as the Child Behavior Checklist, has shown a disappointing performance in our patient population. The BRIEF has been translated and is presently being used by the neuropsychological assessment team at QECH with promising early findings. This is an 86 question instrument with versions for both school age and preschool age children adding about 15 minutes to the evaluations.

For adding renal evaluations

Since the clinical trial linked to this study began, a growing body of literature has begun to elucidate a connection between acute kidney injury (AKI), such as can occur with CM,[40] and the subsequent risk of chronic kidney injury.[41] Ibuprofen may contribute to renal injury. As such, there are stopping rules within the FEVER RCT protocol and data safety and monitoring plan to moderate these risks and the annual interim analysis (two conducted to date) specifically includes an unblinded analysis aimed at determining if there is evidence of excess renal injury in children allocated to the active study drug arm that includes ibuprofen. In this follow-up observational study of children who have completed the FEVER study, we are seeking to add renal function assessments to determine if there is evidence of chronic kidney disease (CKD) in CM survivors enrolled in FEVER and whether CKD is associated with study treatment allocation. This question is made even more relevant by a recent small clinical trial showing acetaminophen to be provide renal protection in malaria thought to be mediated by its ability to inhibit injurious plasma hemoglobin mediated oxidation.[42]

For SARS-Cov-2 testing

Cerebral malaria brain injury occurs in the setting of a proinflammatory cytokine cascade [43] [8], increased excitotoxicity[44, 45], refractory seizures mediated by excitotoxic neurotransmitters[44, 45], and acceleration of free radical production.[46, 47], [48] Severe acute respiratory syndrome coronavirus 2 (*SARS-CoV-2*) down regulates the angiotensin converting enzyme-2 receptor causing over activation of the renin-angiotensin system and producing disruption in vasodilation, neuroinflammation, oxidative stress and thrombotic response.[49] Consequently, malaria coinfections with SARS-CoV-2 could modify disease severity, worsen neurologic injuries and outcome and/or shift the age-susceptibility of malaria disease.[50] If the inflammatory milieu of SARS-Co-V2 further contributes to the endothelial dysfunction and blood brain barrier disruption associated with CM, this might contribute to prolonged parasitemia, more prolonged coma, increased cerebral edema (and thus higher mortality rates)[51], more severe and refractory seizures, and a greater burden of neurological sequelae including epilepsy. A SARS-Cov-2 test is done at discharge from the acute admission when child is still not 100% cognitively recovered. Assent is sought when the child returns for an MRI after the child is fully recovered and can participate in an assent process.

Risk and Benefits

Adding the BRIEF to the neurobehavioral outcomes assessments increases the evaluation time by about 15 minutes and thus potentially increases annoyance and inconvenience This has been added to the consent form.

Adding the renal assessments requires a finger prick and urine sample. Any child found to have evidence of CKD will be notified and an appropriate referral made to assure clinical follow-up is in place. Monitoring is indicated to assure timely initiation of antihypertensive treatment if needed. Careful documentation in the child' medical record and in family education would also be undertaken so that renal toxic medications can be

avoided with any subsequent illness. Data regarding CKD outcomes will be provided to the FEVER Study for inclusion in interim analyses as well.

We are seeking to obtain a swab for SARS-Cov-2 testing around the time of enrollment. SARS-CoV-2 screening is not routinely conducted in the inpatient pediatric settings where FEVER is being conducted, there are no specific treatment for COVID and universal precautions with masking etc. are already in place. As such, there is little benefit to the patient to testing although notification that the child is positive may allow the family to make more informed decisions regarding behaviors and exposures. SARS-Cov-2 testing will be an option aspect of the consent.

Addendum IV.

Proposed Protocol Amendment version 6.1 to 7.0

Amendment changes proposed

1. Sedation changes/clarification
    a. In Malawi, melatonin will no longer be used for sedation. Children will be provided with a snack.
    b. In Zambia, if children are imaged at Medland Hospital and sedation is needed, this will be managed by their consultant anesthesiologist responsible for sedation
2. In Zambia only, families will have the option of participating in an expanded study of epileptogenesis and autism that includes
    a. At enrollment, 10 question developmental screen and additional questions regarding past CNS injuries
    b. An EEG at the 1-and 12-month scheduled MRI Ancillary study follow-up.
    c. Additional follow-up at 6, 18 and 24 months' post recovery from CM which will include an EEG, an epilepsy screening questionnaire, gross motor function classification system clinical assessment, neurological examination and EEG

Rationale

For sedation changes/clarifications

IN MALAWI

As per protocol, when sedation is needed, the Malawi team has used melatonin as the first line medication. They report that melatonin has been almost universally ineffective in their children especially for those children who have behavioral issues evident post CM. This then requires (as per protocol) administration of the second drug planned for sedation—chloral hydrate. Given the general failure of melatonin to provide sufficient sedation for MRI completion in the Malawian setting and the delays and inconvenience for the family when two drugs given in series are needed, melatonin will no longer be used for MRI sedation. If sedation is needed, chloral hydrate (the drug listed as second line in the original protocol) will be used.

IN ZAMBIA

Given technical and scheduling challenges at the Cancer Disease Hospital (CDH) and the need to assure that research patients do not displace imaging for clinical patients, imaging may occur at Medland Hospital. This contingency (i.e. imaging at private facilities) was outlined in the original approved protocol. Understandably, Medland Hospital policies prohibit non-Medland staff from administering sedation to their patients. As such, should sedation be needed for children imaged at Medland Hospital, this would be managed by the Consultant Anesthesiologist for Medland with the understanding that if light sedation with an oral medication is not sufficient, imaging will be deferred. No children will be required to have intravenous or injectable medications.

AT BOTH SITES

Children are often arriving for their follow-up and imaging evaluations having eaten nothing before an early departure from home. As such, we will provide food for them before their evaluations.

For expanded study of epileptogenesis and autism at Chipata Central Hospital only

After recovery from CM, ~10% of children will develop epilepsy although this will usually occur more than 12 months after recovery and is thus an outcome that will not be captured in the MRI Ancillary Study which has only 1- and 12-month follow-ups.[5] Within the context of this prospective cohort study, adding longer-term

follow-up could allow this important outcome to be identified. Additional funding through NINDS has been sought to support longer term follow-up with additional assessments at 6, 12, 18 and 24-months post CM recovery. A separate consent will be sought for this longer term study which will also address two additional scientific questions.

Scientific query 1: Is the autism spectrum disorder (ASD) part of the neurodevelopmental problems that afflict children who have survived CM? It is well-established that CM places surviving children at risk of motor, sensory and language deficits.[5] Whether they are also at risk of autism remains unknown. Autism is increasingly recognized as being among the contributors to childhood developmental problems globally and in Zambia. [52] Understanding whether CM-related brain injury is contributing to this burden would help inform public policy and care. To identify the development of autism and other contextual neurodevelopmental problems among children post CM recovery, this expanded study will include:

At baseline—the 10 questions screen and additional queries re past CNS injuries
At 6, 12, 18 and 24-months follow-up—expanded neurodevelopmental evaluation with the 23-Questions Developmental Assessment (23Q), the Gross Motor Function Classification System (GMFCS) clinical assessment, and a neurological examination.

Scientific query 2: Can quantitative analysis of serial EEG data identify before the development of epilepsy those post CM children who will ultimately development epilepsy? Although 10% of children who survive CM can be expected to develop epilepsy, resource limited setting often do not have sufficient resources to provide ongoing follow-up and monitoring of all children who have recovered from severe malaria. Understandably, families are also often reluctant to continue regular follow-ups for a child who appears entirely well. Being able to recognize children who are going to develop epilepsy based upon EEG findings would allow for improvements in care and might inform future neuroprotective clinical trials. Identifying EEG changes that occur in association with epileptogenesis would also make substantial contributions to our understanding of this process. Professor Birbeck (C-PI for the MRI Ancillary study) in collaboration with Dr. Archana Patel (child neurologist and epileptologist affiliated with Zambia's Paediatric Centre of Excellence and Boston Children's Hospital), recently showed that quantitative analysis of acute EEG obtained with a standard scalp (i.e. non-invasive) recording offers some capacity to predict which children will go on to develop epilepsy. [53] We propose to use these same quantitative analytic methods applied to the EEGs obtained during the acute CM admission and then at 1, 6, 12, 18 and 24 months while also completing a brief epilepsy screening questionnaire to identify incident epilepsy. Hence the expanded study will include:

Epilepsy screening questionnaire and routine EEG at the 1, 6, 12, 18, and 24-month follow-up

<u>Risk and Benefits</u>
All evaluations completed as part of this expanded study of autism and epileptogenesis are non-invasive. No biological samples are required. While the data collection instruments used for this expansion include data such as admission quantitative parasite count during acute CM, this is data already being collected in the primary study and is not, in fact, novel information being collected for this expansion only.

Adding the additional follow-ups at 6, 18 and 24 months requires time and effort from the family. A separate informed consent process will be undertaken and families may opt out of this longer term study but remain in the MRI Ancillary study with the shorter period of follow-up. Families will be compensated for their round trip travel as well as refreshments during their visit. All the same measures will be in place to protect the family's privacy—all data will be entered into RedCap with only the study subject ID (no names). Paper-based records with identifiers will remain on site in locked study offices accessible only to research staff, all of whom have undergone training in the protection of human subjects.

The expanded neurodevelopmental assessments and epilepsy screening tool may facilitate for a more expedient diagnosis of seizure disorders and/or developmental problems in children as they are recovering from CM. Identifying these problems earlier will allow for more rapid treatment including antiseizure

medications to prevent seizures, status epilepticus and seizure-related injuries, counseling of families regarding safety measures to prevent seizure-related injuries, communications with schools to encourage retention of children in school if behavioral issues limit their full engagement in school, and individualized therapy for children based upon their developmental challenges. Dr. Patel has substantial experience in this realm including at the PCOE DIC Clinic. Zambian staff have already undergone training to conduct the neurodevelopmental evaluations and during Dr. Patel's time in Zambia (some months each year) she will further train staff in therapeutics. Essentially, care for any conditions identified over the course of the study will be provided through the facility where the work is being conducted. Epilepsy care services are already available at all study facilities and additional EEGs if needed for clinical care can be acquired.

References

1. Carter JA, Lees JA, Gona JK, et al. Severe falciparum malaria and acquired childhood language disorder. Dev Med Child Neurol 2006;48:51-57.
2. Carter JA, Mung'ala-Odera V, Neville BG, et al. Persistent neurocognitive impairments associated with severe falciparum malaria in Kenyan children. J Neurol Neurosurg Psychiatry 2005;76:476-481.
3. Carter JA, Neville BG, White S, et al. Increased prevalence of epilepsy associated with severe falciparum malaria in children. Epilepsia 2004;45:978-981.
4. Dondorp AM, Fanello CI, Hendriksen IC, et al. Artesunate versus quinine in the treatment of severe falciparum malaria in African children (AQUAMAT): an open-label, randomised trial. Lancet 2011;376:1647-1657.
5. Birbeck GL, Molyneux ME, Kaplan PW, et al. Blantyre Malaria Project Epilepsy Study (BMPES) of neurological outcomes in retinopathy-positive paediatric cerebral malaria survivors: a prospective cohort study. Lancet Neurol 2010;9:1173-1181.
6. Carter JA, Ross AJ, Neville BG, et al. Developmental impairments following severe falciparum malaria in children. Trop Med Int Health 2005;10:3-10.
7. Idro R, Jenkins NE, Newton CR. Pathogenesis, clinical features, and neurological outcome of cerebral malaria. Lancet Neurol 2005;4:827-840.
8. John CC, Bangirana P, Byarugaba J, et al. Cerebral malaria in children is associated with long-term cognitive impairment. Pediatrics 2008;122:e92-99.
9. Severe and complicated malaria. World Health Organization, Division of Control of Tropical Diseases [see comments]. Trans R Soc Trop Med Hyg 1990;84:1-65.
10. Corbett D, Thornhill J. Temperature modulation (hypothermic and hyperthermic conditions) and its influence on histological and behavioral outcomes following cerebral ischemia. Brain Pathol 2000;10:145-152.
11. Greer DM, Funk SE, Reaven NL, Ouzounelli M, Uman GC. Impact of fever on outcome in patients with stroke and neurologic injury: a comprehensive meta-analysis. Stroke 2008;39:3029-3035.
12. Laptook AR, Corbett RJ. The effects of temperature on hypoxic-ischemic brain injury. Clin Perinatol 2002;29:623-649, vi.
13. Suz P, Vavilala MS, Souter M, Muangman S, Lam AM. Clinical features of fever associated with poor outcome in severe pediatric traumatic brain injury. J Neurosurg Anesthesiol 2006;18:5-10.
14. Arrich J, Holzer M, Herkner H, Mullner M. Cochrane corner: hypothermia for neuroprotection in adults after cardiopulmonary resuscitation. Anesth Analg 2010;110:1239.
15. Lin ZL, Yu HM, Lin J, Chen SQ, Liang ZQ, Zhang ZY. Mild hypothermia via selective head cooling as neuroprotective therapy in term neonates with perinatal asphyxia: an experience from a single neonatal intensive care unit. J Perinatol 2006;26:180-184.
16. Low E, Boylan GB, Mathieson SR, et al. Cooling and seizure burden in term neonates: an observational study. Arch Dis Child Fetal Neonatal Ed 2012;97:F267-272.
17. Schulzke SM, Rao S, Patole SK. A systematic review of cooling for neuroprotection in neonates with hypoxic ischemic encephalopathy - are we there yet? BMC Pediatr 2007;7:30.
18. Smit E, Liu X, Jary S, Cowan F, Thoresen M. Cooling neonates who do not fulfil the standard cooling criteria - short and long term outcomes. Acta paediatrica 2014.

19. Choi HA, Badjatia N, Mayer SA. Hypothermia for acute brain injury--mechanisms and practical aspects. Nat Rev Neurol 2012;8:214-222.
20. Corry JJ. Use of hypothermia in the intensive care unit. World J Crit Care Med 2012;1:106-122.
21. Magnusson M, Dahl M, Cederberg J, Karlsson M, Sandstrom R. Pharmacodynamics of Carbamazepine-mediated Induction of CYP3A4, CYP1A2, and Pgp as Assessed by Probe Substrates Midazolam,Caffeine, and Digoxin. Clinical Pharmacology & Therapeutics 2008;84:52-62.
22. Scaravilli V, Tinchero G, Citerio G, Participants in the International Multi-Disciplinary Consensus Conference on the Critical Care Management of Subarachnoid H. Fever management in SAH. Neurocritical care 2011;15:287-294.
23. Chomba E, Haworth A, Atadzhanov M, Mbewe E, Birbeck GL. The socioeconomic status of children with epilepsy in Zambia: implications for long-term health and well-being. Epilepsy Behav 2008;13:620-623.
24. Crawley J, Smith S, Kirkham F, Muthinji P, Waruiru C, Marsh K. Seizures and status epilepticus in childhood cerebral malaria. Qjm 1996;89:591-597.
25. Dammann O, Leviton A. Inflammation, brain damage and visual dysfunction in preterm infants. Semin Fetal Neonatal Med 2006;11:363-368.
26. Moxon CA, Chisala NV, Wassmer SC, et al. Persistent endothelial activation and inflammation after Plasmodium falciparum Infection in Malawian children. The Journal of infectious diseases 2014;209:610-615.
27. Langfitt JT, McDermott MP, Brim R, et al. Neurodevelopmental Impairments 1 Year After Cerebral Malaria. Pediatrics 2019.
28. Potchen MJ, Kampondeni SD, Seydel KB, et al. Acute Brain MRI Findings in 120 Malawian Children with Cerebral Malaria: New Insights into an Ancient Disease. AJNR Am J Neuroradiol 2012;10.3174/ajnr.A3035.
29. Pongponratn E, Riganti M, Punpoowong B, Aikawa M. Microvascular sequestration of parasitized erythrocytes in human falciparum malaria: a pathological study. Am J Trop Med Hyg 1991;44:168-175.
30. Taylor TE, Fu WJ, Carr RA, et al. Differentiating the pathologies of cerebral malaria by postmortem parasite counts. Nat Med 2004;10:143-145.
31. Medhi N, Das SB, Das RR, et al. MRI findings of cerebral malaria. A report of two cases. Neuroradiol J 2009;22:407-412.
32. Rasalkar DD, Paunipagar BK, Sanghvi D, Sonawane BD, Loniker P. Magnetic resonance imaging in cerebral malaria: a report of four cases. Br J Radiol 2011;84:380-385.
33. Potchen MJ, Kampondeni SD, Ibrahim K, et al. NeuroInterp: a method for facilitating neuroimaging research on cerebral malaria. Neurology 2013;81:585-588.
34. Kampondeni SD, Birbeck GL, Seydel KB, et al. Noninvasive measures of brain edema predict outcome in pediatric cerebral malaria. Surg Neurol Int 2018;9:53.
35. Shu L, Zhong K, Chen N, et al. Predicting the severity of white matter lesions among patients with cerebrovascular risk factors based on retinal images and clinical laboratory data: a deep learning study. Front Neurol 2023;14:1168836.
36. Ibekwe R, Jeaven L, Wilmshurst JM. The role of melatonin to attain electroencephalograms in children in a sub-Saharan African setting. Seizure 2017;51:87-94.
37. Gordon SB, Chinula L, Chilima B, et al. A Malawi guideline for research study participant remuneration. Wellcome Open Res 2018;3:141.
38. D'Agostino RB, Jr. Propensity score methods for bias reduction in the comparison of a treatment to a non-randomized control group. Stat Med 1998;17:2265-2281.
39. Brim R, Mboma S, Semrud-Clikeman M, et al. Cognitive Outcomes and Psychiatric Symptoms of Retinopathy-Positive Cerebral Malaria: Cohort Description and Baseline Results. Am J Trop Med Hyg 2017;97:225-231.
40. Plewes K, Turner GDH, Dondorp AM. Pathophysiology, clinical presentation, and treatment of coma and acute kidney injury complicating falciparum malaria. Curr Opin Infect Dis 2018;31:69-77.
41. Conroy AL, Opoka RO, Bangirana P, et al. Acute kidney injury is associated with impaired cognition and chronic kidney disease in a prospective cohort of children with severe malaria. BMC Med 2019;17:98.
42. Plewes K, Kingston HWF, Ghose A, et al. Acetaminophen as a Renoprotective Adjunctive Treatment in Patients With Severe and Moderately Severe Falciparum Malaria: A Randomized, Controlled, Open-Label Trial. Clin Infect Dis 2018;67:991-999.

43. John CC, Park GS, Sam-Agudu N, Opoka RO, Boivin MJ. Elevated serum levels of IL-1ra in children with Plasmodium falciparum malaria are associated with increased severity of disease. Cytokine 2008;41:204-208.
44. Dobbie M, Crawley J, Waruiru C, Marsh K, Surtees R. Cerebrospinal fluid studies in children with cerebral malaria: an excitotoxic mechanism? The American journal of tropical medicine and hygiene 2000;62:284-290.
45. Medana IM, Day NP, Salahifar-Sabet H, et al. Metabolites of the kynurenine pathway of tryptophan metabolism in the cerebrospinal fluid of Malawian children with malaria. The Journal of infectious diseases 2003;188:844-849.
46. Becker K, Rahlfs S, Nickel C, Schirmer RH. Glutathione--functions and metabolism in the malarial parasite Plasmodium falciparum. Biological chemistry 2003;384:551-566.
47. Greve B, Lehman LG, Lell B, Luckner D, Schmidt-Ott R, Kremsner PG. High oxygen radical production is associated with fast parasite clearance in children with Plasmodium falciparum malaria. The Journal of infectious diseases 1999;179:1584-1586.
48. Clark IA, Mackie EJ, Cowden WB. Injection of free radical generators causes premature onset of tissue damage in malaria-infected mice. The Journal of pathology 1986;148:301-305.
49. Divani AA, Andalib S, Di Napoli M, et al. Coronavirus Disease 2019 and Stroke: Clinical Manifestations and Pathophysiological Insights. J Stroke Cerebrovasc Dis 2020;29:104941.
50. Gutman JR, Lucchi NW, Cantey PT, et al. Malaria and Parasitic Neglected Tropical Diseases: Potential Syndemics with COVID-19? The American journal of tropical medicine and hygiene 2020.
51. Seydel KB, Kampondeni SD, Valim C, et al. Brain swelling and death in children with cerebral malaria. N Engl J Med 2015;372:1126-1137.
52. Davids EL, Adams Tucker L, Wambua GN, et al. Child and adolescent mental health in Africa: A qualitative analysis of the perspectives of emerging mental health clinicians and researchers using an online platform. J Child Adolesc Ment Health 2019;31:93-107.
53. Patel AA, Jannati A, Dhamne SC, et al. EEG markers predictive of epilepsy risk in pediatric cerebral malaria - A feasibility study. Epilepsy Behav 2020;113:107536.
