# Supplementary material for: Protocol for a magnetic resonance imaging study of participants in the fever RCT: Does fever control prevent brain injury in malaria?
Source: PLoS One. 2024 Apr 19;19(4):e0294823. doi: 10.1371/journal.pone.0294823 (PMC11029645; doi:10.1371/journal.pone.0294823)
Supplement: S2 File — (PDF) [file pone.0294823.s003.pdf]

UNIVERSITY of ROCHESTER

APPROVAL

December 2, 2020

The RSRB approved the following submission from 12/1/2020 to 11/2/2021 inclusive. This approval is effective as of 12/1/2020. This study is multi-site and the RSRB is the IRB of Record for a Non-UR site. Additional review and approval is required of the Non-UR site.

Type of Review:	Initial Study
Title:	An MRI Ancillary Study of a Malaria Fever Randomized Controlled Trial (RCT)
Investigator:	Gretchen Birbeck
Study ID:	STUDY00005384
Funding:	National Institute of Neurological Disorders and Stroke (NINDS)
Regulatory Findings Consent:	Parent permission obtained and documented
Regulatory Findings HIPAA:	Waiver or alteration of HIPAA
Documents Reviewed:	2 MRI Ancillary Assent Form English Version 3.0 10Nov2020 , Chipata Hospital Letter of Support, MRI Ancillary Consent Form English Version 2.0 to 3.1 tracked 19Nov2020.pdf, QECH letter of support.pdf, Request for Non-UR Site to Rely on UR RSRB MSU (1).docx, Malawi COMREC approval letter, Data Collection Instruments V2.0.pdf, Data Security Agreement 2October2020.docx, MRI Ancillary to Fever RCT Protocol V4.0 to 5.1 tracked.pdf

This study was reviewed and approved under the OHSP and UR policies, and in accordance with Federal regulation 45 CFR 46 under the University's Federal-wide Assurance (FWA00009386). As the Principal Investigator, you are responsible for ensuring compliance with Policy 901 Investigator Responsibilities. Click here for the Summary of Responsibilities for Investigators Conducting Non-FDA Regulated Research. Also, any unanticipated problems involving risks to subjects or others (including unexpected deaths, hospitalizations or serious injuries, breach of confidentiality, loss of privacy) must be reported according to Policy 801 Reporting Research Events.

Association for the Accreditation of Human Research Protection Programs, Inc. Full AAHRPP Accreditation
